# Enzymatic Logic of Ubiquitin Chain Assembly

**DOI:** 10.3389/fphys.2019.00835

**Published:** 2019-07-05

**Authors:** Kirandeep K. Deol, Sonja Lorenz, Eric R. Strieter

**Affiliations:** ^1^Department of Chemistry, University of Massachusetts, Amherst, MA, United States; ^2^Rudolf Virchow Center for Experimental Biomedicine, University of Würzburg, Würzburg, Germany; ^3^Department of Biochemistry and Molecular Biology, University of Massachusetts, Amherst, MA, United States

**Keywords:** ubiquitin, E2 conjugating enzyme, E3 ligating enzyme, sequential addition, en bloc transfer

## Abstract

Protein ubiquitination impacts virtually every biochemical pathway in eukaryotic cells. The fate of a ubiquitinated protein is largely dictated by the type of ubiquitin modification with which it is decorated, including a large variety of polymeric chains. As a result, there have been intense efforts over the last two decades to dissect the molecular details underlying the synthesis of ubiquitin chains by ubiquitin-conjugating (E2) enzymes and ubiquitin ligases (E3s). In this review, we highlight these advances. We discuss the evidence in support of the alternative models of transferring one ubiquitin at a time to a growing substrate-linked chain (sequential addition model) versus transferring a pre-assembled ubiquitin chain (en bloc model) to a substrate. Against this backdrop, we outline emerging principles of chain assembly: multisite interactions, distinct mechanisms of chain initiation and elongation, optimal positioning of ubiquitin molecules that are ultimately conjugated to each other, and substrate-assisted catalysis. Understanding the enzymatic logic of ubiquitin chain assembly has important biomedical implications, as the misregulation of many E2s and E3s and associated perturbations in ubiquitin chain formation contribute to human disease. The resurgent interest in bifunctional small molecules targeting pathogenic proteins to specific E3s for polyubiquitination and subsequent degradation provides an additional incentive to define the mechanisms responsible for efficient and specific chain synthesis and harness them for therapeutic benefit.

## Introduction

**FIGURE 1:**
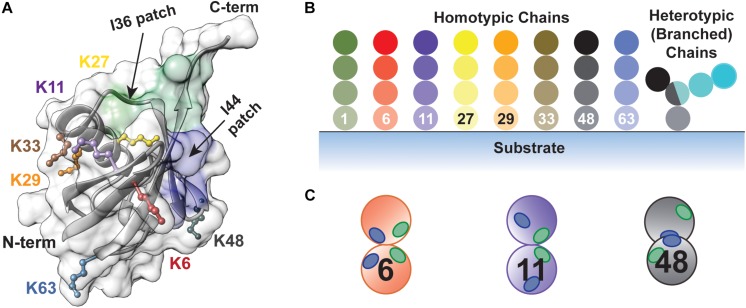
The diversity of ubiquitin chains enables highly nuanced mechanisms of regulation. **(A)** Structure of ubiquitin (PDB: 1UBQ) showing the seven lysine side chains (K6, K11, K27, K29, K33, K48, and K63) and the N-terminus along with the I36 and I44-centered hydrophobic patches, highlighted in green (L8, I36, L70, and L73) and blue (L8, I44, H68, and V70), respectively. **(B)** Types of ubiquitin chains that can be produced. Homotypic chains are assembled using the same amino group of ubiquitin for each linkage, whereas heterotypic chains contain a mixture of linkages. Branched chains are a form of heterotypic chain, in which at least one ubiquitin molecule is modified by two or more ubiquitin molecules. **(C)** The relative orientation of the I36 and I44-centered hydrophobic patches controls how different chain types are recognized by effector proteins and DUBs.

The small protein ubiquitin is involved in nearly every cellular pathway through its covalent attachment to target proteins (Hershko and Ciechanover, 1998; Oh et al., 2018). Ubiquitination is notorious for triggering protein degradation through the 26S proteasome (Finley, 2009; Collins and Goldberg, 2017; Yu and Matouschek, 2017). However, it can also alter protein structure and function, induce changes in protein localization, and mediate the assembly or disassembly of multi-protein complexes. What determines the fate of a ubiquitinated protein is the nature of the modification (Komander and Rape, 2012; Swatek and Komander, 2016; Yau and Rape, 2016). Ubiquitin molecules can be covalently attached to a target protein at one or several sites to afford a (multi-) monoubiquitinated product and/or in the form of polyubiquitin chains. Owing to the eight amino groups of ubiquitin (M1, K6, K11, K27, K29, K33, K48, and K63) along with the capacity to form branched structures, the number of possible chain types is staggering; each one has the potential to govern the dynamics of a biochemical pathway in a distinct manner (Figure 1). Due to the diverse functional consequences, there has been intense interest in deciphering how ubiquitin chains are assembled.

Over a decade ago, a thought-provoking review was published describing the state of affairs regarding ubiquitin chain assembly (Hochstrasser, 2006). At the time, several models had been proposed, but the precise mechanisms by which ubiquitin chains are generated on a substrate by the sequential activities of E1 (ubiquitin-activating), E2 (ubiquitin-conjugating), and E3 enzymes (ubiquitin ligases) remained unclear. Since then, the picture has become much less opaque: detailed kinetic studies on several E2-E3 systems have supported a sequential addition mechanism, in which ubiquitin molecules are added one at a time, initially to the substrate and then to the distal end of the growing chain (Pierce et al., 2009; Lu et al., 2015b; French et al., 2017). In certain cases, chain initiation and elongation are performed by two distinct E2 enzymes working in collaboration with a single E3 (Rodrigo-Brenni and Morgan, 2007; Garnett et al., 2009; Williamson et al., 2009; Wu K. et al., 2010; Wu T. et al., 2010). In other instances, both steps are carried out by distinct pairs of E2s and E3s (Scott et al., 2016; Dove et al., 2017a). With some systems there is also evidence for an “en bloc” transfer of pre-assembled ubiquitin chains to substrates (Wang and Pickart, 2005; Li et al., 2007; Ravid and Hochstrasser, 2007; Masuda et al., 2012; Ronchi et al., 2013, 2017; Streich et al., 2013; Edwards et al., 2014; Todaro et al., 2017).

In this review, we focus on the mechanistic intricacies of ubiquitin chain formation. We start by providing a census of the ubiquitination/deubiquitination machinery to underscore the diversity of enzymes involved. We then revisit mechanisms of chain assembly that have been put forward over the years, providing detailed accounts in support of each one. Finally, we outline the key factors underlying the efficiency, processivity, and specificity with which E2s and E3s catalyze chain assembly.

## Cellular Machinery

**FIGURE 2:**
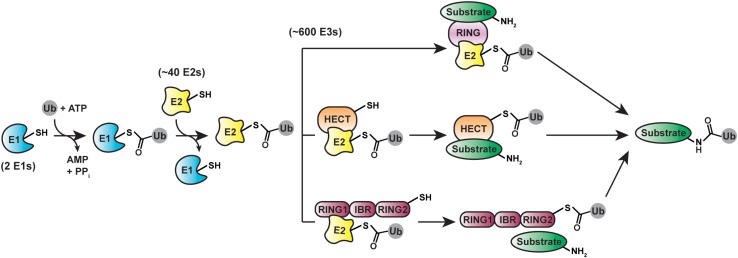
Cellular machinery involved in ubiquitination. protein ubiquitination is driven by E1 (ubiquitin-activating), E2 (ubiquitin-conjugating), and E3 (ubiquitin-ligating) enzymes. Three distinct classes of ubiquitin ligases—RING/U-box (top), HECT (middle), and RBR (bottom)—utilize different structural mechanisms for mediating the final transfer of ubiquitin onto substrates.

A massive collection of proteins encoded by ∼5% of the human genome is responsible for sculpting the cellular ubiquitination landscape (Clague et al., 2015; Figure 2). The apex of the system is the E1 family of enzymes (Schulman and Wade Harper, 2009). In humans, there are two E1s selective for ubiquitin: UBA1 and UBA6. UBA1 is one of the most abundant protein in HeLa cells (Kulak et al., 2014). UBA6—which is an order of magnitude lower in abundance than UBA1—is unique in that it loads ubiquitin or the ubiquitin-like modifier FAT10 specifically onto the E2 UBE2Z (USE1), whereas UBA1 transfers ubiquitin to a wide array of E2s (Chiu et al., 2007; Jin et al., 2007; Pelzer et al., 2007; Aichem et al., 2010). The human genome encodes ∼40 E2s dedicated to ubiquitin conjugation (Michelle et al., 2009; Yates et al., 2017). Several of them are limited to monoubiquitination; others act as “chain extenders” by modifying ubiquitin itself. A subset of E2s synthesize a single linkage type between ubiquitin molecules, while others are promiscuous (Ye and Rape, 2009; Wenzel et al., 2015; Stewart et al., 2016).

By selecting substrates and modifying them with ubiquitin chains, E3s play a pivotal role in ubiquitin signaling. Considering most proteins in the cell are subject to ubiquitination, the substrate repertoire of E3s is immense. To meet this demand, the human genome encodes over 600 E3s (Li et al., 2008), which fall into three mechanistic classes: the RING (really interesting new gene)/U-box, HECT (homologous to E6AP C-terminus), and RBR (RING-between-RING) ligases (Buetow and Huang, 2016). The RING/U-box ligases are the largest of these classes and catalyze the direct transfer of ubiquitin from a thioester-linked E2-ubiquitin conjugate (E2∼ubiquitin) to a substrate (Deshaies and Joazeiro, 2009; Metzger et al., 2014). The 28 human HECT (Huibregtse et al., 1995; Lorenz, 2018; Weber et al., 2019) and 14 RBR (Marín et al., 2004; Smit and Sixma, 2014; Dove and Klevit, 2017; Walden and Rittinger, 2018) ligases proceed through a two-step mechanism, in which ubiquitin is first transferred from an E2 to the active site cysteine of the E3 to afford an E3∼ubiquitin thioester-linked intermediate and then delivered to a substrate (Huang et al., 1999; Wenzel et al., 2011).

The ∼100 human deubiquitinases (DUBs) antagonize the activity of E3s by severing the isopeptide linkage between ubiquitin and a substrate or disassembling ubiquitin chains (Clague et al., 2013, 2019; Mevissen and Komander, 2017). DUBs are sub-categorized into seven families: the ubiquitin C-terminal hydrolases (UCHs), ubiquitin specific proteases (USPs), Machado-Josephins (MJDs), ovarian tumor proteases (OTUs), JAB1/MPN domain-associated metalloisopeptidases (JAMM/MPN^+^), the novel MIU-containing DUB family MINDY (Abdul Rehman et al., 2016), and the zinc finger with UFM1-specific peptidase domain-containing ZUFSP family (Haahr et al., 2018; Hermanns et al., 2018; Hewings et al., 2018; Kwasna et al., 2018). All but the JAMM/MPN^+^ metalloproteases catalyze bond cleavage using an active-site cysteine residue reminiscent of canonical cysteine proteases such as papain. Proteins containing ubiquitin-binding domains (UBDs) typically act as effectors transmitting the recognition of different ubiquitin modifications into a biological response (Husnjak and Dikic, 2012).

## What Are the Different Mechanisms of Chain Assembly?

Several models for chain assembly have been entertained over the years (Hochstrasser, 2006). While these models involve different oligomerization states of E2s and E3s, they all follow either of two basic mechanisms—sequential addition and en bloc transfer—which differ in the directionality of chain growth and location of the growing chain. Sequential addition—presumably the predominant mechanism—requires the transfer of individual ubiquitin molecules to a substrate. By contrast, the en bloc mechanism involves transferring chains that have been pre-formed on the active-site cysteine of an E2 or HECT/RBR E3 to a substrate.

### Sequential Addition

**FIGURE 3:**
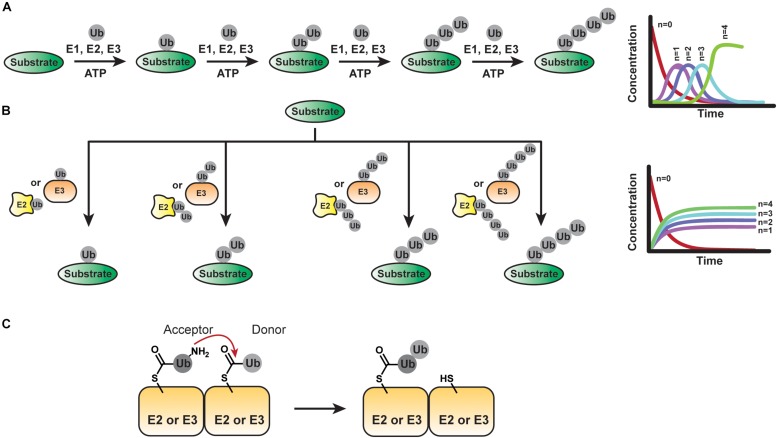
Mechanisms of Ubiquitin Chain Assembly. **(A)** The sequential addition model implies the transfer of ubiquitin molecules one at a time. The fractional conversion profile of product formation will show a lag phase proportional to chain length for each new ubiquitinated species formed. **(B)** In the en bloc model, pre-assembled ubiquitin chains anchored to the active site of an E2 or E3 are transferred to a substrate. If the pre-assembly of chains is much faster than the transfer of chains to the substrate, a lag phase proportional to chain length should not be observed in the fractional conversion profile. **(C)** Basic step of an en bloc transfer mechanism: The ubiquitin conjugated to the active site of an E2 or HECT/RBR E3 provides an acceptor lysine residue to attack the C-terminus of another thioester-linked ubiquitin tethered to another E2 or E3.

According to the sequential addition model, each ubiquitinated substrate species acts as a substrate for the formation of successively longer substrate-linked chain (Figure 3A). Thus, new ubiquitinated species appear in a sequential manner with a lag phase proportional to chain length. The challenge in providing evidence for this mechanism lies in the necessity to detect individual reaction products/intermediates on fast timescales of milliseconds to seconds.

This was first accomplished using the yeast Skp-cullin-F-box protein complex SCF^Cdc4^ (human homolog of Cdc4 is FBXW7) as a model system (Pierce et al., 2009). SCF^Cdc4^ is the founding member (Feldman et al., 1997; Skowyra et al., 1997) of the largest family of E3s—the cullin-RING ligases (CRLs) (Petroski and Deshaies, 2005a)—which account for ∼20% of all proteasome-dependent degradation (Soucy et al., 2009). It cooperates with the K48 linkage-specific E2 Cdc34 (human enzyme is referred to as UBE2R1) (Petroski and Deshaies, 2005b; Gazdoiu et al., 2007; Ziemba et al., 2013; Choi et al., 2014; Chong et al., 2014; Hill et al., 2016). Together, SCF^Cdc4^ and Cdc34 catalyze substrate polyubiquitination within seconds. Using single-encounter assays, it was shown that most encounters of Cdc34-SCF^Cdc4^ with a substrate are futile (Pierce et al., 2009); however, the vast majority of modified substrate molecules carry chains with ≥4 ubiquitin moieties (Saha and Deshaies, 2008). To determine whether substrate ubiquitination by Cdc34-SCF^Cdc4^ occurs en bloc or sequentially, the distribution of ubiquitin chains conjugated to Cdc34 was measured by intact mass spectrometry (Pierce et al., 2009); the logic being that the product distribution should reflect the population of Cdc34∼ubiquitin conjugates if pre-formed chains were transferred from the E2 to the substrate en bloc. However, Cdc34 conjugates bearing more than one ubiquitin could not be detected in this set-up, thus offering indirect support for a sequential addition mechanism.

The most compelling evidence for the sequential addition mechanism comes from millisecond kinetic measurements (Pierce et al., 2009). Such time resolution is sufficient to detect whether chains are formed sequentially or contemporaneously. In the case of Cdc34-SCF^Cdc4^ and UBE2R1-SCF^β— TrCP^, each new ubiquitinated species was found to occur sequentially with non-concurrent lag phases; in other words, each reaction intermediate acts as a substrate during each round of chain elongation. Kinetic analyses revealed that transferring the first ubiquitin (a step represented by *k*_Ub1_) is the slowest step during ubiquitin chain assembly, which is why most enzyme-substrate encounters are unproductive. Once the first ubiquitin is in place, the rates of sequential ubiquitin additions (*k*_Ub__–__n_) are markedly faster than substrate dissociation (*k*_off_), allowing for multiple transfer events to occur before the enzyme-substrate complex dissociates. As a result, polyubiquitination is processive. Just as the probability of acquiring a chain depends on the ratio of *k*_Ub1_ to *k*_off_, the length of a chain is determined by the rates of the additional ubiquitin transfer (*k*_Ub__–__n_) and *k*_off_. Reductions in *k*_Ub__–__n_ manifest with increasing chain length, as the distal end of the growing chain samples more conformational space.

With certain E3s, e.g., the 1.3 MDa CRL APC/C, the conformational space occupied by the growing chain can be restricted by subunits harboring ubiquitin-binding domains (Brown et al., 2014; Kelly et al., 2014), resulting in a decrease in *k*_off_ and an increase in *k*_Ub__–__n_. As described in more detail below, single-molecule measurements combined with detailed structural and biochemical studies have shown that the APC/C assembles ubiquitin chains through a feed-forward mechanism termed processive affinity amplification (PAA) (Lu et al., 2015b). The basic premise is that substrates carrying more ubiquitin molecules/longer ubiquitin chains are preferentially ubiquitinated compared to substrates carrying fewer ubiquitin molecules/shorter chains. In essence, each ubiquitin molecule added to the substrate enhances the affinity of the modified substrate for the APC/C, thus rendering chain formation processive.

A consequence of the sequential addition mechanism is that the processivity of ubiquitin linkage formation can dictate chain length and, thus, the fate of the substrate protein. In the case of the Cdc34/UBE2R1-SCF complexes, the rate-limiting transfer of the first ubiquitin affords two distinct populations of substrate–unmodified and extensively polyubiquitinated (Pierce et al., 2009). How fast a substrate is decorated with the first ubiquitin will thus largely determine how fast it is degraded by the proteasome, assuming ubiquitination is rate-limiting during degradation.

The situation is different with the HECT ligase WWP1. Time course data shows that WWP1 catalyzes K63-linked ubiquitin chains on its substrates in a sequential manner with a lag phase proportional to chain length (French et al., 2017). Once the chain reaches ∼4 subunits in length, WWP1 switches to building chains linked through K11 and K48, presumably due to topological constraints on the distal ubiquitin within the substrate-tethered K63 chain. Thus, even if the multidirectional phase is slow, the substrate can be modified with short K63 chains to possibly direct proteasome-independent events. In contrast, branched chains, as formed during the second phase of the reaction promote proteasomal degradation of the modified substrate (Meyer and Rape, 2014; Grice et al., 2015; Liu et al., 2017; Yau et al., 2017; Ohtake et al., 2018).

Sequential addition also lends itself to fine-tuning of the polyubiquitinated protein by DUBs (Zhang et al., 2013): consider two substrates with slightly different affinities for an E3. Both acquire ubiquitin moieties at roughly the same rate, but one is released faster than the other. Every time dissociation occurs, the growing ubiquitin chain may be exposed to a DUB, thus running the risk of disassembly. Indeed, incorporating DUB activity into a kinetic model for sequential addition reveals that a two-fold increase in *k*_off_ results in over an eight-fold decrease in chain formation. In other words, modest differences in E3-substrate affinity afford significant differences in the extent to which substrates are modified with ubiquitin in a single encounter with the E3. In turn, this affects the efficiency of their proteasome-mediated degradation or alternative downstream responses.

### En Bloc Transfer

In addition to transferring individual ubiquitin molecules to a growing substrate-linked chain, there is evidence that certain systems can pre-form chains on the active site cysteine of an E2 or E3 before transfer to a substrate (Figure 3B). From the perspective of maximizing the efficiency with which a substrate is polyubiquitinated, such en bloc transfer is ideal: unlike the sequential addition mechanism, an E3-substrate complex does not have to be long-lived for the substrate to receive a chain of sufficient length for downstream signaling events. Instead, the population of E2- or E3-tethered chains dictates how a substrate is modified. On the flip side, en bloc transfer requires the mechanisms of chain pre-formation on the respective enzyme to be much faster than substrate transfer.

En bloc transfer was first proposed based on biochemical studies with the K48-specific HECT E3 UBE3A (E6AP) (Wang et al., 2006). As with all HECT E3s, ubiquitin is transferred from an E2 (UBE2L3) to the active-site cysteine of UBE3A to form a thioester-linked conjugate. En bloc assembly of chains by UBE3A then implies that the ubiquitin conjugated to the active site of UBE3A provides the acceptor lysine residue (K48) to attack the C-terminus of another thioester-linked ubiquitin tethered to either a UBE3A subunit (in the context of an UBE3A oligomer) or an associated E2 (Figure 3C). Consistent with this notion, mixing UBE3A∼ubiquitin with a ubiquitin molecule that cannot be activated as a thioester precludes the formation of di-ubiquitin. However, di-ubiquitin can be generated upon reacting UBE3A∼ubiquitin with an E2∼K0-ubiquitin conjugate (ubiquitin variant lacking lysine residues). When a substrate is added to the picture, e.g., the ubiquitin-binding protein HHR23A, the K48-linked di-ubiquitin is transferred to HHR23A, suggesting en bloc transfer is chemically feasible. However, UBE3A-bound di-(or poly)ubiquitin has not been detected directly. Recently, steady-state kinetic analyses have led to a model in which E2∼ubiquitin conjugates bind two functionally distinct sites on trimers of the catalytic domain of UBE3A (Ronchi et al., 2013, 2014, 2017) and NEDD4 subfamily members (Todaro et al., 2017, 2018), respectively, to build active site-anchored ubiquitin chains for en bloc transfer. Yet, as mentioned above, the NEDD4-type WWP1 builds chains sequentially (French et al., 2017).

Time course analyses suggested that en bloc transfer also applies to certain E2/RING E3 systems. The human E2 UBE2G2 (Fang et al., 2001; Chen et al., 2006) and its yeast ortholog Ubc7 (Biederer et al., 1996; Hiller et al., 1996; Bays et al., 2001; Deak and Wolf, 2001) are associated with the ER membrane and responsible for K48-linked ubiquitin chain formation on misfolded polypeptides exported from the ER lumen during ERAD. In the presence of the ER-resident E3 GP78, UBE2G2 was found to catalyze the assembly of ubiquitin chains on its active-site cysteine (Li et al., 2007). The propensity for GP78 to oligomerize drives this preassembly process, as the formation of UBE2G2-GP78 hetero-oligomers brings the active sites of multiple UBE2G2 molecules into close proximity (Li et al., 2009; Liu et al., 2014). With UBE2G2∼ubiquitin conjugates juxtaposed, K48 of one ubiquitin is thought to attack the C-terminus of the neighboring thioester-linked one.

Besides UBE2G2, UBE2K, and Ubc7, have also been found conjugated to ubiquitin chains through a thioester linkage (Haldeman et al., 1997; Ravid and Hochstrasser, 2007; Bazirgan and Hampton, 2008). The rate at which chains are formed on the active-site cysteine of UBE2G2 is slightly faster than the rate of transfer to a substrate lysine residue, suggesting en bloc transfer is kinetically feasible during the polyubiquitination of a substrate (Li et al., 2007). That said, fast kinetic measurements of product distributions would be helpful to validate this mechanism and confirm that substrate-anchored ubiquitin chains indeed reflect the distribution of chains attached to the active site of UBE2G2.

## What Are the Key Molecular Features Required for Chain Assembly?

Ubiquitin chain formation requires a molecular juggling act (Lorenz et al., 2013). In the context of the sequential addition model, an E3 has to engage a substrate and a thioester-linked E2-donor ubiquitin complex, transfer ubiquitin to a primary amino group of the substrate, and then switch to one of eight primary amino groups of an acceptor ubiquitin to promote chain elongation. All of these events must occur while the substrate remains bound to the E3 to minimize the number of encounters required to build a chain.

### Multisite E2-E3 Interactions During Chain Formation

**FIGURE 4:**
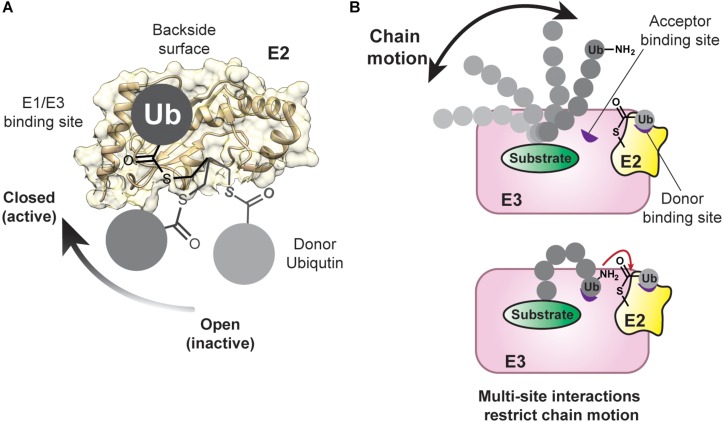
Conformational Flexibility of the Donor and Acceptor Ubiquitin Are Restrained During Chain Assembly. **(A)** The donor ubiquitin covalently tethered to the active-site cysteine of an E2 can adopt multiple conformations, referred to as “open” and “closed.” The closed conformation is important during catalysis with RING E3 enzymes. The E1/E3 binding site and the backside of an E2 are also highlighted on the structure of UBE2D2 (PDB: 5ULF). **(B)** As a chain increases in length, it can occupy more conformational space (top). Low-affinity ubiquitin-binding domains (shown in purple) can assist in limiting the conformational space of the chain and positioning the acceptor ubiquitin for the nucleophilic attack on the E2∼donor ubiquitin conjugate.

Structural studies have shown that E3s interact with the same surface of E2s that is recognized by E1 enzymes (Figure 4A; Eletr et al., 2005; Huang et al., 2005; Kamadurai et al., 2009; Lechtenberg et al., 2016; Dove et al., 2017b; Yuan et al., 2017; Condos et al., 2018; Sauvé et al., 2018). Such mutually exclusive binding places a major constraint on the mechanism of polyubiquitination because the spent E2 must dissociate from the E3 after ubiquitin transfer to allow for ubiquitin reloading. Assuming the association between E3 and an E2∼ubiquitin conjugate is governed by short-range interactions (e.g., van der Waals), and thus diffusion-controlled, the on-rate would be ≤10^6^ M^–1^s^–1^ (Alsallaq and Zhou, 2008; Schreiber et al., 2009). With an intracellular concentration of E2∼ubiquitin conjugate estimated to be around 1 μM (Kleiger et al., 2009a), the complex would form at a rate of 1 s^–1^. If the affinity is ∼10 μM, the off-rate would be 10 s^–1^. For six rounds of conjugation, the assembly and disassembly of an E2-E3 complex would thus consume ∼6 s of the entire process. At first glance, these numbers seem reasonable, considering kinetic studies have shown that a substrate can acquire up to six ubiquitin molecules in ∼10 s, as catalyzed by the Cdc34-SCF complex (Saha and Deshaies, 2008). However, Cdc34∼ubiquitin binds tightly to SCF with a *K*_d_ in the low nanomolar range (Saha and Deshaies, 2008). The overall rate of cyclical binding and release would thus not be conducive to the processive assembly of a ubiquitin chain.

A balance between affinity and processivity is achieved through bipartite interactions. Cdc34 not only engages SCF through the Rbx1 RING subunit, but also possesses an acidic C-terminal tail that interacts with a conserved basic cleft on the cullin subunit (Kleiger et al., 2009b). These electrostatic interactions govern the initial, rapid recognition, while interactions between Rbx1 and Cdc34 position and activate the ubiquitin-loaded E2 for transferring ubiquitin to the substrate. With electrostatic forces driving the interaction between Cdc34 and SCF, the on-rate is two orders of magnitude faster than the diffusion-controlled limit. The off-rate thus does not have to be slow in order to achieve nanomolar affinity. Since the off-rate is >30 s^–1^, binding and release can occur before substrate dissociation. That electrostatic forces govern the interactions between Cdc34 and the SCF has an additional advantage: due to their weaker dependence on distance (1/*r*) compared to van-der-Waals interactions (1/*r*^6^) allows the spent E2 to remain in close proximity to the E3 while it is recharged with ubiquitin by an E1. The importance of electrostatic forces in Cdc34-CRL interactions is underscored by the fact that the basic cleft is conserved across the cullin family.

Other multi-component E2-CRL assemblies have also evolved multisite binding mechanisms for processive polyubiquitination. For example, the human APC/C cooperates with two E2s, UBE2C and UBE2S, to catalyze polyubiquitination of key cell cycle regulators, thereby regulating cellular progression through mitosis—a process that has recently been reconstructed, primarily due to the power of cryo-electron microscopy (Alfieri et al., 2017; Watson et al., 2019). UBE2C initiates chain synthesis, while UBE2S catalyzes chain elongation in a K11-linkage specific manner (Jin et al., 2008; Garnett et al., 2009; Williamson et al., 2009; Wu T. et al., 2010; Wickliffe et al., 2011). The rate ot substrate degradation is determined by the efficiency of chain initiation as well as the nature and extent of polyubiquitination (Rape et al., 2006; Williamson et al., 2011; Lu et al., 2015a). Processivity in ubiquitination, as achieved through the PAA mechanism discussed above (Lu et al., 2015b), ensures efficient substrate degradation and involves distinct multisite interactions of the APC/C with UBE2C and UBE2S.

During the priming reaction, the APC/C engages UBE2C in a canonical mode through its RING domain (APC11) and via the winged-helix B (WHB) domain of APC2 (Brown et al., 2014, 2015, 2016). The WHB domain binds the “backside” of UBE2C, which is located on the opposite side of the donor ubiquitin binding site and provides an important allosteric site in a number of E2s (Miura et al., 2002; Brzovic et al., 2006; Bocik et al., 2011; Hibbert et al., 2011; Page et al., 2012; Ranaweera and Yang, 2013). Investigations into another RING E3, RNF38, and the E2 UBE2D2 (Buetow et al., 2015) have shown that backside binding of ubiquitin to UBE2D2 limits the flexibility of the RING domain binding site and stabilizes the catalytically active closed conformation of UBE2D2∼ubiquitin (Figure 4A; Saha et al., 2011; Wickliffe et al., 2011; Dou et al., 2012, 2013; Plechanovová et al., 2012; Pruneda et al., 2012; Soss et al., 2013; Branigan et al., 2015; Dove et al., 2017b). The net result is a dramatic increase in catalytic efficiency. Based on these data, the WHB domain of APC2 might not only limit the search radius of the dynamic UBE2C-APC11 assembly to specific lysines of a substrate (Brown et al., 2015), but also potentiate ubiquitin transfer.

In other systems, backside interactions enable rapid cycling between E2 binding and release. For example, the G2BR domain of GP78 was found to bind to the backside of UBE2G2 with low nanomolar affinity (Das et al., 2009; Li et al., 2009). This interaction induces conformational changes in UBE2G2 that result in an increase in the affinity for the RING domain of GP78 by ∼50-fold. Assuming the binding energies are additive (Jencks, 1981), an overall *K*_d_ in the picomolar range would be expected. Such tight binding could adversely affect E2 exchange from the E3 and ultimately processivity; however, the measured *K*_d_ turns out to be ∼10^3^ times higher than calculated. As revealed through biophysical studies, UBE2G2 is released from GP78 by an allosteric feedback mechanism in which binding of the RING domain to UBE2G2 reduces the number of electrostatic interactions between UBE2G2 and G2BR (Das et al., 2013). The kinetics in this system are such that the off-rate of UBE2G2 from G2BR is much faster than the rate of ubiquitin transfer. Since the E1 cannot charge the G2BR-bound form of UBE2G2 as efficiently as free UBE2G2, complete release of the E2 is likely important for consecutive cycles of ubiquitination.

Chain elongation on APC/C substrates involves a unique set of bipartite interactions between the APC/C and UBE2S. UBE2S is primarily recruited to APC/C through its 66-residue C-terminal peptide (CTP) extension (Meyer and Rape, 2014; Brown et al., 2016), which nestles into a complementary acidic and hydrophobic groove at the interface between the APC2 and APC4 subunits. The catalytic UBC domain of UBE2S is held in place by the cullin APC2, not the APC11 RING domain, to promote chain elongation (Brown et al., 2016). The APC11 RING domain instead serves a non-canonical role during this process by tracking the distal acceptor ubiquitin molecule of the growing ubiquitin chain (Kelly et al., 2014; Brown et al., 2016).

### Distinct Enzymes for Priming and Extension

The distinct reactions of monoubiquitination/priming and polyubiquitination/elongation that are catalyzed by E3s present significant challenges. During the priming phase, ubiquitin is directly attached to a substrate protein. For this process to be site-specific, spatial restrictions (Kamadurai et al., 2013) or active participation from the substrate (Jin et al., 2008; Mattiroli et al., 2012, 2014) are required. In most cases monoubiquitination occurs at multiple lysines with little dependence on sequence context (Petroski and Deshaies, 2003; Tang et al., 2007; Fischer et al., 2011). By contrast, chain elongation often occurs with specificity for the amino group of ubiquitin that is modified (Wenzel and Klevit, 2012). To achieve specificity during sequential addition, an E3 needs to repeatedly position the distal acceptor ubiquitin molecule in a growing chain with high precision.

E3s have evolved different mechanisms to meet this demand. The APC/C, for example, uses two different E2s for priming and extension (Rodrigo-Brenni and Morgan, 2007). The recognition of a substrate’s degron motif (a KEN- or D-box) by the APC/C co-factors CDH1 or CDC20, respectively, along with the APC10 subunit (Fang et al., 1998; Pfleger and Kirschner, 2000) places the substrate in close proximity to the UBE2C∼ubiquitin conjugate (Brown et al., 2016). Confined to the same space within the central cavity of APC/C, UBE2C can readily modify the substrate with individual ubiquitin molecules and/or short K11, K48, and K63 chains (Kirkpatrick et al., 2006). Thereafter, the APC/C subsequently juxtaposes UBE2S with the acceptor ubiquitin (Brown et al., 2016), thus providing an optimal geometry for processive and specific chain elongation using K11 of ubiquitin (Wickliffe et al., 2011). What is particularly interesting about the different catalytic architectures of the APC/C is the potential for differential regulation by macromolecular factors and posttranslational modifications, e.g., phosphorylation (Reimann et al., 2001; Craney et al., 2016; Qiao et al., 2016).

Similar to the APC/C, SCF ligases are known to collaborate with several E2s. In this case, UBE2D2/D3 transfers the first ubiquitin, while UBE2R1 catalyzes K48-linked chain elongation (Wu K. et al., 2010). In a surprising turn of events, SCFs were also found to team up with another E3 ligase to carry out the distinct steps of priming and extension (Scott et al., 2016; Dove et al., 2017a). Using genome and proteome-wide screens, the RBR E3 HHARI and its *Caenorhabditis elegans* ortholog ARI-1 were shown to associate with the NEDD8-modified/activated forms of CRLs. Like many RBRs, HHARI/ARI-1 exists in an autoinhibited state in which its Ariadne domain interacts with the RING2 domain (Duda et al., 2013), thereby blocking the catalytic cysteine and preventing transfer of ubiquitin from the cognate E2 UBE2L3/UBC-18. Autoinhibition is relieved upon binding of NEDD8-modified CRLs. Once charged with ubiquitin, HHARI catalyzes the monoubiquitination of CRL substrates. With the substrate primed, UBE2R1 then works with the CRL to form a K48-linked chain. While this cooperative mode of action of HHARI and NEDD8-modified CRLs is conceptually unique, there are other examples of ubiquitin ligases that regulate each other through macromolecular interfaces (Kühnle et al., 2011).

When two E3s act independently but sequentially to prime and extend, the ubiquitin-dependent outcome can change dramatically. In response to replication fork collapse, for example, the DNA polymerase processivity factor PCNA (proliferating cell nuclear antigen) is monoubiquitinated by Rad6 (an E2) and Rad18 (an E3) to promote an error-prone damage tolerance process known as translesion DNA synthesis (Hoege et al., 2002; Stelter and Ulrich, 2003). Switching to an error-free, lesion bypass process requires collaboration between the E3 Rad5 and E2 Ubc13/Mms2 to build K63-linked chains, with the monoubiquitinated product of Rad6-Rad16 serving as the substrate for Ubc13/Mms2-Rad5 (Hoege et al., 2002; Parker and Ulrich, 2009). The HECT E3s HUWE1 and UBR5 also use ubiquitinated substrates to seed chain extension. HUWE1 has been shown to recognize substrates primed with K63-linked chains by another E3, e.g., TRAF6, and extend a K48-linked chain from a branch point (Ohtake et al., 2016), transforming the ubiquitin-dependent signal from non-degradative to degradative. Likewise, UBR5 installs K48 branch points on preexisting chains built by other E3s, e.g., ITCH and UBR4, and continues to extend the chains with K48 linkages for the purpose of creating a more potent proteasome-targeting signal (Yau et al., 2017; Ohtake et al., 2018). Whether HUWE1 and UBR5 prefer an internal ubiquitin subunit within a chain, similar to the yeast branching enzyme Ufd2 (Liu et al., 2017), or any subunit can serve as the starting point for chain extension remains unclear.

### Orienting the Acceptor Ubiquitin

While the orientation of the donor ubiquitin toward the E2 or E3 is an important part of preparing the active site for the nucleophilic attack of the acceptor, both chemically and sterically (Figure 4A; Saha et al., 2011; Wickliffe et al., 2011; Dou et al., 2012, 2013; Plechanovová et al., 2012; Pruneda et al., 2012; Soss et al., 2013; Branigan et al., 2015; Lorenz et al., 2016; Dove et al., 2017b), the acceptor ubiquitin must also be positioned properly to maximize the efficiency and processivity of chain formation (Figure 4B; Wright et al., 2016). One can think about this problem from an entropic perspective: as the length increases, a chain can occupy more conformational space. To avoid a large entropic penalty, E2s and E3s must limit the conformational freedom of a growing chain by placing the acceptor ubiquitin in close proximity to the donor. Importantly, how an acceptor ubiquitin is oriented toward the donor also determines the specificity of linkage formation.

The first clue for how an acceptor ubiquitin is positioned for the formation of a specific linkage came from structural studies of the K63-specific yeast E2 Ubc13 (human enzyme; UBE2N) and its co-factor, the ubiquitin E2 variant (UEV) Mms2 (UBE2V2). A crystal structure of the Mms2-Ubc13-donor ubiquitin conjugate showed the donor ubiquitin of one complex binding to the Mms2 molecule of a neighboring Mms2-Ubc13 complex (Eddins et al., 2006). The resulting contacts between Mms2 and ubiquitin position K63 near the active site of the adjacent Ubc13, mimicking an acceptor ubiquitin. This structure thus illustrated how an accessory protein can assist in limiting the orientation of the acceptor ubiquitin toward the catalytic center of an E2 and allow for specificity in acceptor lysine selection. More recently, the crystal structures of a UBE2V2/UBE2N-donor ubiquitin complex bound to the dimeric RING domain of RNF4 revealed that the RING domain tethers the donor ubiquitin in an activated closed conformation toward the E2, thus stimulating catalysis (Branigan et al., 2015); this mechanism has emerged as a canonical principle of RING-mediated catalysis in several E2/E2 systems (Dou et al., 2012; Plechanovová et al., 2012; Pruneda et al., 2012).

By contrast, the APC/C relies on a non-canonical interaction between its RING domain (APC11) and the acceptor ubiquitin: a hydrophobic patch of APC11 engages ubiquitin through residues surrounding K48 along with the C-terminus, thus blocking K48 from serving as an acceptor lysine (Brown et al., 2016), and allowing K11 to be presented to the activated C-terminus of the UBE2S-linked donor. Kinetic studies suggest this mechanism accounts for a 40-fold decrease in *K*_m_ and an overall 175-fold increase in catalytic efficiency (Brown et al., 2016).

Interestingly, UBE2S can achieve K11 linkage specificity in the absence of the APC/C (Bremm et al., 2010; Matsumoto et al., 2010; Wickliffe et al., 2011). The key to this inherent specificity lies in the activation of the acceptor lysine, K11, by an adjacent acidic side chain, E34, of ubiquitin, which promotes the nucleophilic attack of K11 through facilitating its deprotonation (Wickliffe et al., 2011). This mechanism, known as substrate-assisted catalysis, triggers isopeptide bond formation specifically when K11 is presented to the UBE2S active site, while other acceptor orientations are catalytically disfavored. Notably, ubiquitin was also found to contribute to catalysis in HECT E3s (Ries et al., 2019) and DUBs (Keusekotten et al., 2013; Mevissen et al., 2016), suggesting substrate-assisted catalysis is a conserved theme in different classes of ubiquitinating and deubiquitinating enzymes.

Principles of acceptor ubiquitin recognition have also started to emerge outside of the RING E3 family. For instance, structural studies of the RBR-type linear ubiquitin chain assembly complex (LUBAC) has revealed dedicated interaction sites for the acceptor ubiquitin. LUBAC generates M1-linked chains to regulate innate immunity and inflammation through the NF-κB pathway (Kirisako et al., 2006; Ikeda et al., 2011; Tokunaga et al., 2011) and is composed of two RBR E3 subunits: HOIP and HOIL-1L; however, only one of the RBRs (HOIP), contains the catalytic activity necessary for generating M1-linked chains (Smit et al., 2012; Stieglitz et al., 2013). The structure of a minimal HOIP–ubiquitin transfer complex shows the α-amino group of the acceptor residue, M1, poised for the nucleophilic attack on the donor ubiquitin (Lechtenberg et al., 2016). In this complex, the RING2-domain together with the “linear ubiquitin chain determining region” (LDD) of HOIP create a platform that ensures the α-amino group of the acceptor is positioned in proximity to the active site. Deprotonation of this amino group, as required for its nucleophilic function, is promoted by a particular histidine residue of HOIP.

In HECT E3s, the structural basis of acceptor ubiquitin recognition has remained elusive. However, a particular region, known as the “exosite,” in the N-lobe of the catalytic HECT domain, was shown to engage a regulatory ubiquitin molecule, thus promoting chain elongation but not initiation (Ogunjimi et al., 2010; Kim et al., 2011; Maspero et al., 2011, 2013; Zhang et al., 2016; French et al., 2017; Ries et al., 2019). It has thus been hypothesized that the exosite contributes indirectly to stabilizing the acceptor ubiquitin in proximity to the active site by interacting with flanking ubiquitin moieties within the growing chain (Fajner et al., 2017).

Finally, Cue1, a receptor and activator of the yeast E2 Ubc7 that is crucial for ERAD (Biederer et al., 1997), provides an example of how an accessory protein can impact the acceptor ubiquitin to promote ubiquitin linkage formation. Cue1 harbors an N-terminal ubiquitin-binding CUE domain and a C-terminal Ubc7 binding region (U7BR), which likely activates Ubc7 in a manner analogous to the G2BR domain of GP78 (Bazirgan and Hampton, 2008; Kostova et al., 2009; Metzger et al., 2013). NMR studies and *in vitro* ubiquitination reactions revealed that the CUE domain accelerates chain formation by binding to the penultimate ubiquitin molecule in a growing chain, thereby assisting U7BR in orienting the distal acceptor moiety toward the active site of Ubc*7* formation (Bagola et al., 2013; von Delbrück et al., 2016). Although the overall impact of Cue1 on the kinetics of chain formation is modest *in vitro*, the mechanistic implications are rather intriguing. Kinetic measurements show that the off-rate of the CUE domain-ubiquitin complex is fast compared to the rate of isopeptide bond formation. Thus, the CUE domain can “hop” along a chain without affecting the overall rate of chain formation until it finds the ubiquitin moiety adjacent to the acceptor. E3s capable of elongating existing ubiquitin chains by installing branch points could use similar enzymatic logic (Koegl et al., 1999; Metzger and Weissman, 2010; Ohtake et al., 2016, Ohtake et al., 2018; Liu et al., 2017; Yau et al., 2017).

## Outlook

The progress made over the last decade in understanding how ubiquitin chains are assembled is astonishing. Most notably, detailed kinetic analyses have illustrated the complexities of the sequential addition mechanism in the context of CRLs. Moreover, synergistic advances in structural biology, genomic engineering, quantitative mass spectrometry, and chemical biology have helped elucidate central operating principles underlying the activities of E2s and E3s, including multi-site interactions, the cooperative interplay of distinct enzymes for chain initiation and elongation, the precise positioning of the donor and acceptor ubiquitin, and substrate-assisted catalysis. While these principles have been found to recur in various model systems studied, it is important to realize that each class of E3s appears to implement these principles by distinct structural mechanisms, thus contributing to the enormous versatility and specificity of ubiquitin signaling.

Despite considerable advances in understanding the mechanistic principles of ubiquitin chain formation, reconstituting and structurally visualizing the trajectory of the functional enzyme-substrate assemblies has been challenging. This is due, for the most part, to the weak and dynamic nature of the underlying macromolecular interactions, which typically fall into the hig micromolar to millimolar affinity range *in vitro*. To overcome this challenge, crosslinking approaches have emerged as indispensable tools, opening exciting avenues to capture specific complexes for structural analyses (Witting et al., 2017).

The immense potential of ubiquitin ligases as therapeutic targets has been illustrated by the clinical efficacy of thalidomide and its derivatives in the treatment of hematological malignancies (Lindner and Krönke, 2016). However, progress toward rationally manipulating E3s has been impeded largely by our insufficient understanding of their conformational dynamics, macromolecular interactions, and functional integration into cellular pathways (Huang and Dixit, 2016; Chen et al., 2018). Over the next few years, it will be exciting to see how the mechanisms of critical, yet uncharacterized E3s unfold, especially those in the relatively poorly characterized RBR and HECT families, and how these mechanisms are altered in human diseases. For instance, the HECT ligase HUWE1 has both pro-oncogenic and tumor suppressor functions, depending on the cellular context (Zhao et al., 2008; Inoue et al., 2013; Peter et al., 2014; King et al., 2016; Myant et al., 2017). Which macromolecular complexes mediate these functions by mediating substrate selection, activity, and linkage specificity of this crucial ligase remains to be determined. It will also be important to identify the consequences of patient-derived mutations in disease-associated ligase genes in order to develop efficient strategies targeting these enzymes therapeutically. Finally, a better understanding of the mechanisms of ubiquitin chain formation will facilitate the development of bifunctional small molecules, known as PROTACs (Sakamoto et al., 2002), that re-program a particular ligase to mark a pathogenic target protein for degradation – a powerful concept that has recently entered clinical trials (Mullard, 2019).

## Author Contributions

All authors listed have made a substantial, direct and intellectual contribution to the work, and approved it for publication.

## Conflict of Interest Statement

The authors declare that the research was conducted in the absence of any commercial or financial relationships that could be construed as a potential conflict of interest.
